# Supplementary figures and images for: Assessment of salivary microRNA by RT-qPCR: Facing challenges in data interpretation for clinical diagnosis
Source: PLoS One. 2024 Dec 10;19(12):e0314733. doi: 10.1371/journal.pone.0314733 (PMC11630609; doi:10.1371/journal.pone.0314733)

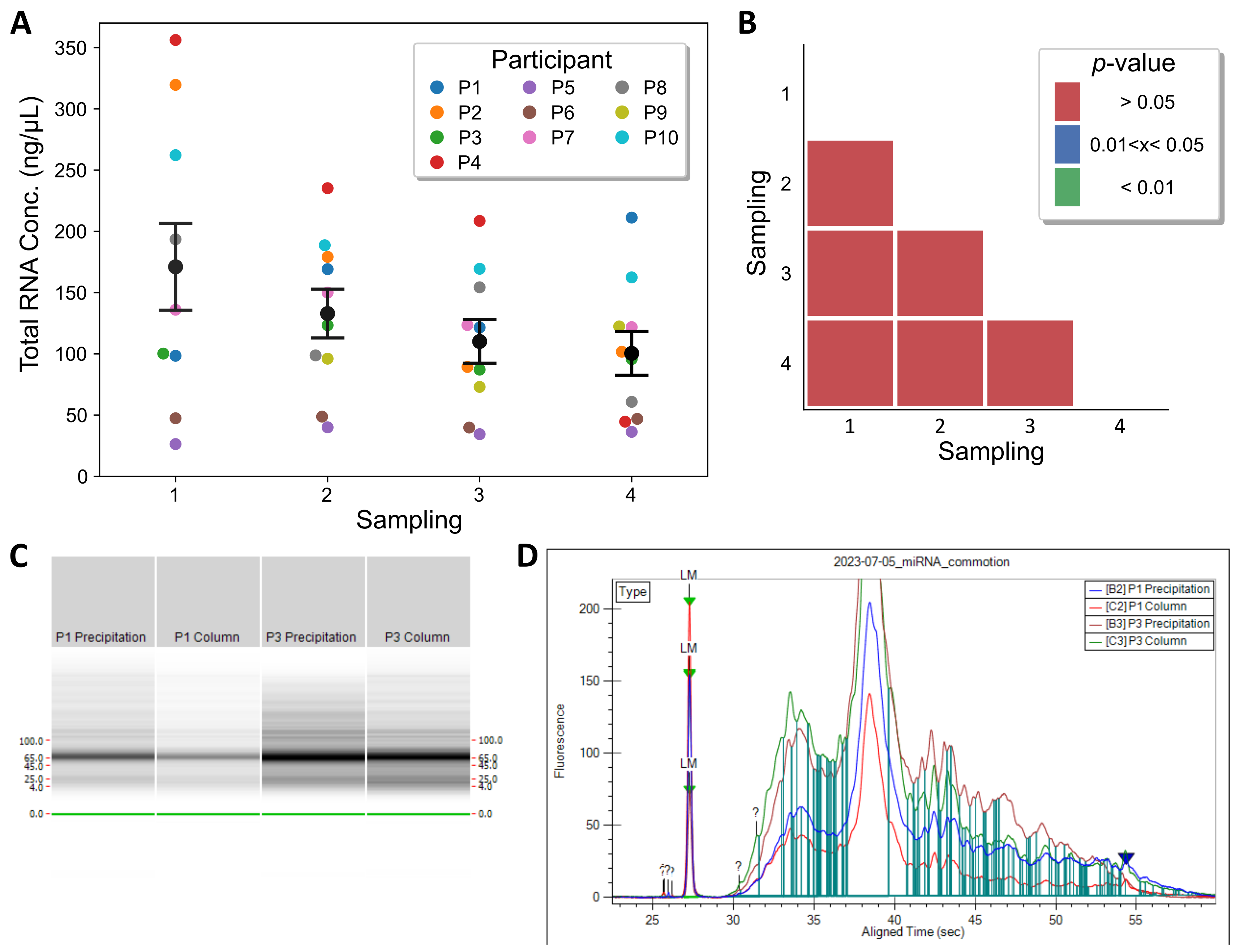

Supplement: S1 Fig — (A) Distribution of the total extracted small RNA concentrations among 10 participants throughout four sampling points. Black points represent the mean value and error bars show the standard error of the mean. (B) Mann-Whitney U results of statistical tests performed on panel A. (C) Gel and (D) electropherogram Labchip data show similar small RNA profiles by two different extraction methods, RNA precipitation or filter column (used in this study). (TIF) [file pone.0314733.s001.tif]

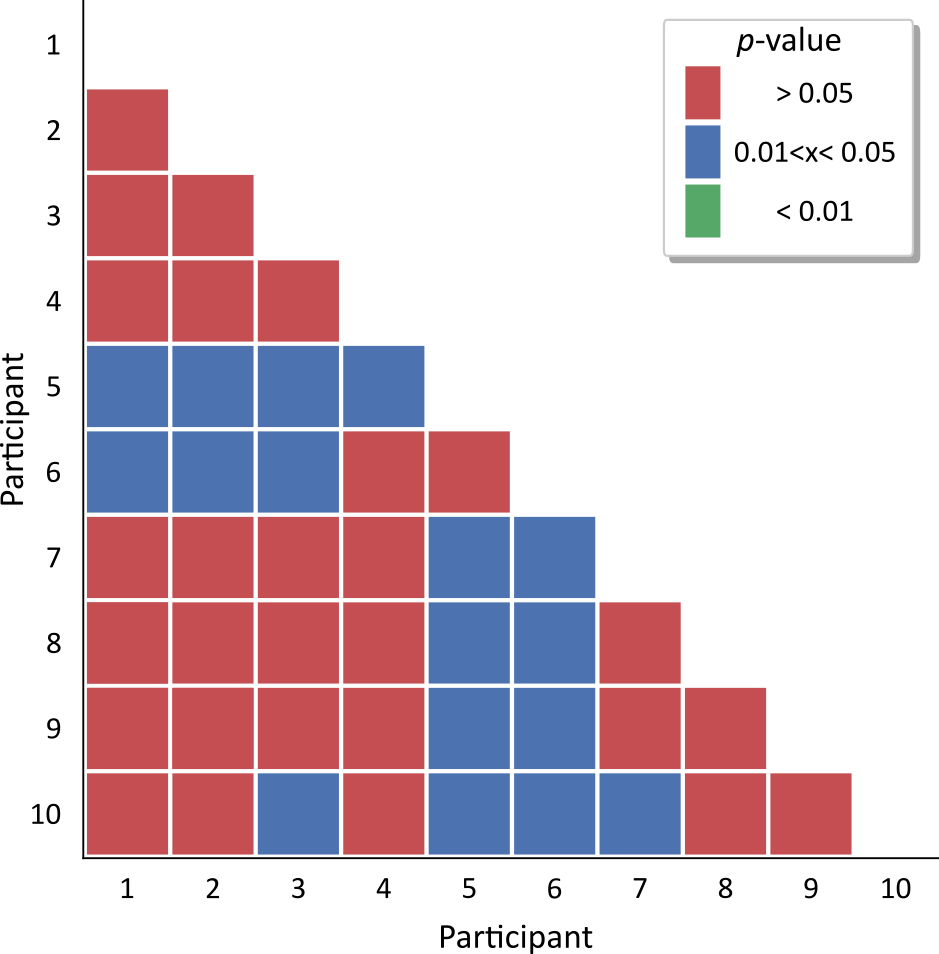

Supplement: S2 Fig — (TIF) [file pone.0314733.s002.tif]

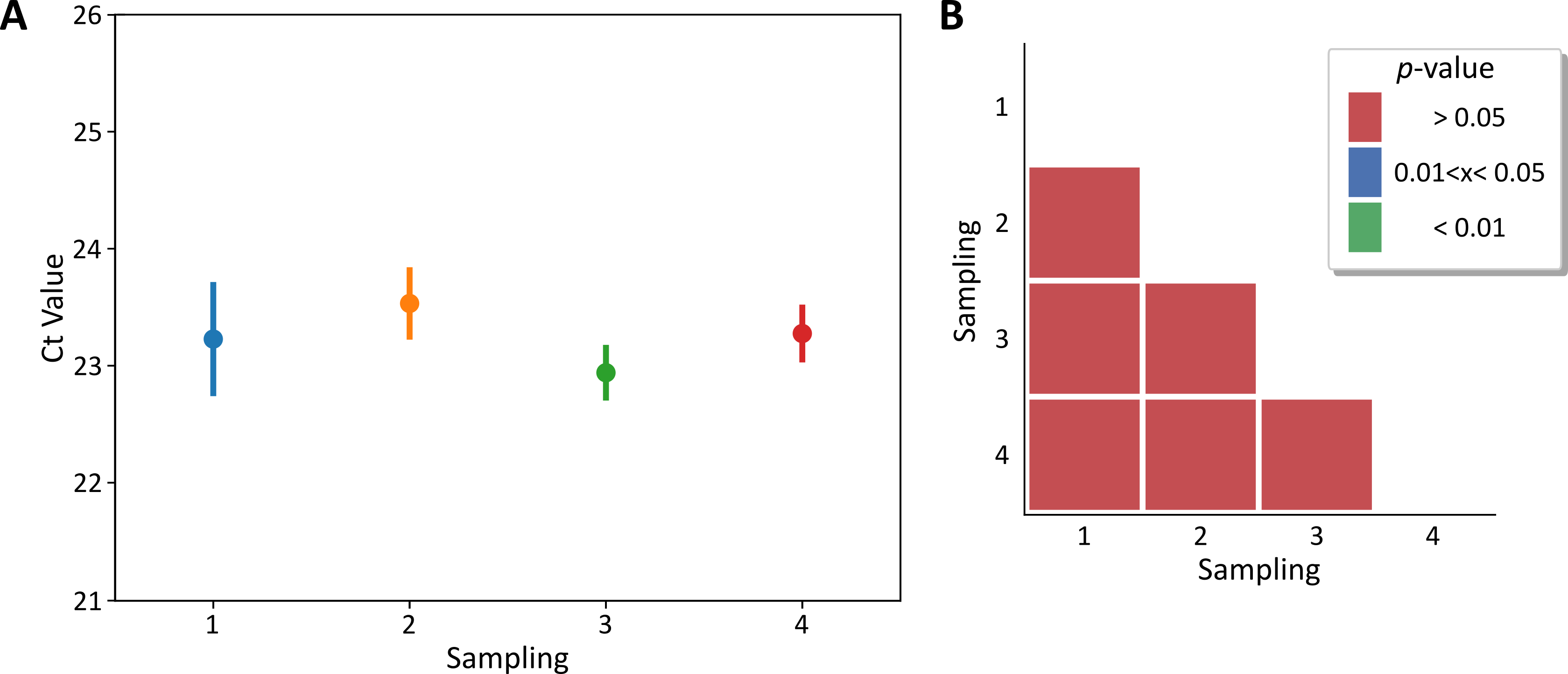

Supplement: S3 Fig — (A) RT-qPCR quantification values of spike-in UniSP6 miRNA using 50 ng of the total extracted salivary small RNAs for the different sampling points. Points represent the mean value and error bars depict the standard error of the mean. (B) Mann-Whitney U results performed on panel A. (TIF) [file pone.0314733.s003.tif]

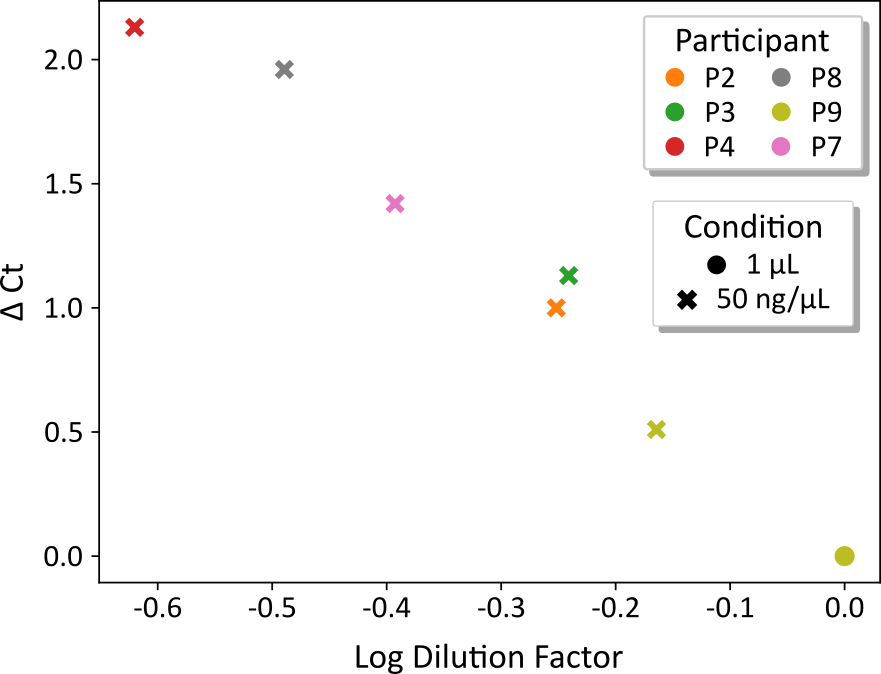

Supplement: S4 Fig — All samples were analysed in two conditions: Using 1μL (circle) or 50 ng (cross) of the total extracted small RNAs as input. Since the same concentration of UniSp6 miRNA is present at 1 μL for all participants, all 50 ng values have been normalized with respect to the RNA concentration at 1 μL (dilution factor), and all Ct values have been shifted with respect to the 1 μL value (causing the overlapping of all 1 μL values). ΔCt values = Ct50ng–Ct1μL. (TIF) [file pone.0314733.s004.tif]

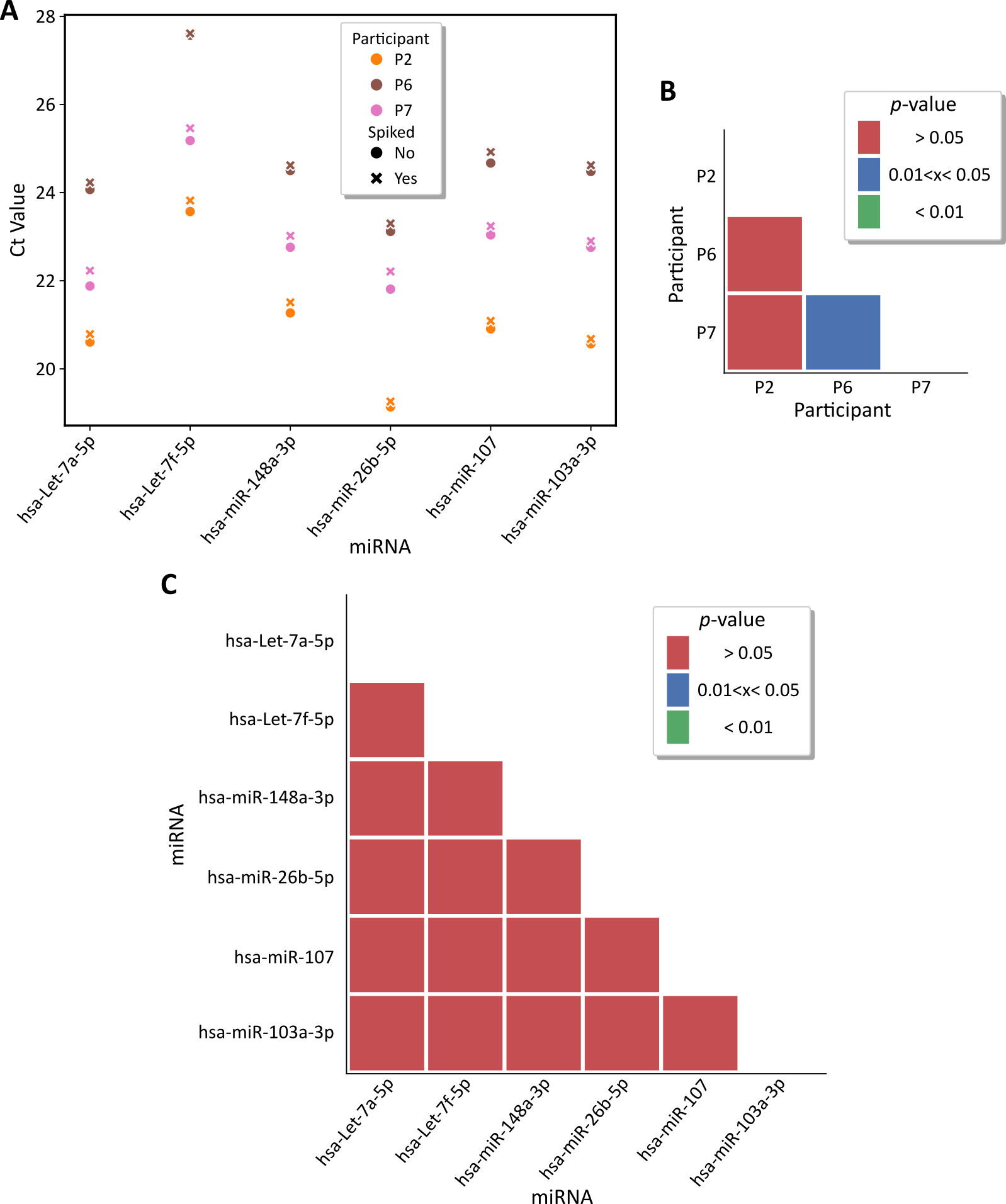

Supplement: S5 Fig — Small RNAs were extracted from three saliva samples (P2, P6 and P7) in the presence and absence of spiked-in artificial UniSP6 miRNA. (A) RT-qPCR quantification for the six miRNAs using 50 ng of small RNAs. Mann-Whitney U results for panel A of ΔCt between with and without spiking with respect to (B) individual participants and (C) miRNA assay. (TIF) [file pone.0314733.s005.tif]

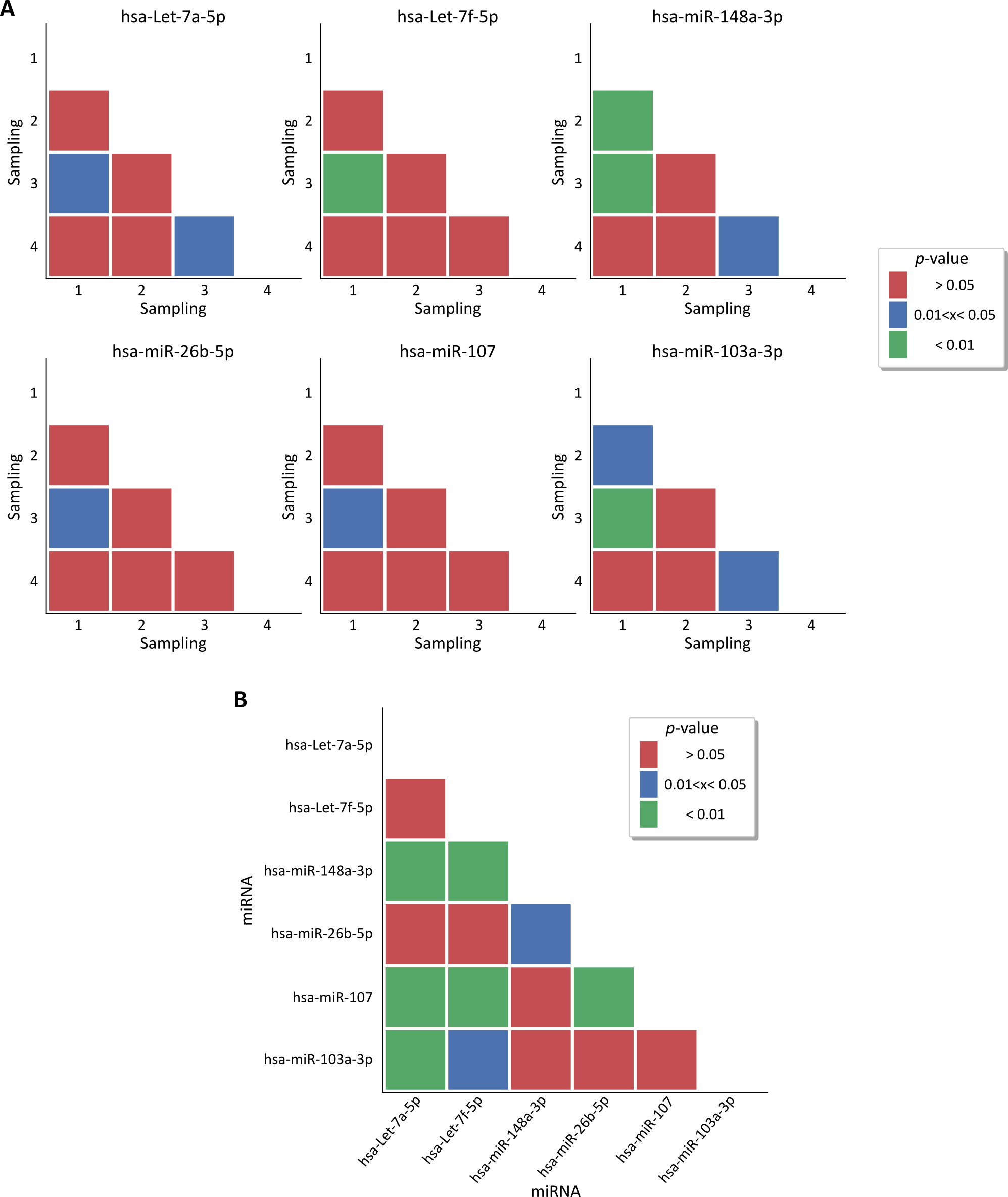

Supplement: S6 Fig — Mann-Whitney U results for data presented in Fig 2B of the manuscript showing differences within (A) samplings for each miRNA assay and (B) within miRNA assays. (TIF) [file pone.0314733.s006.tif]

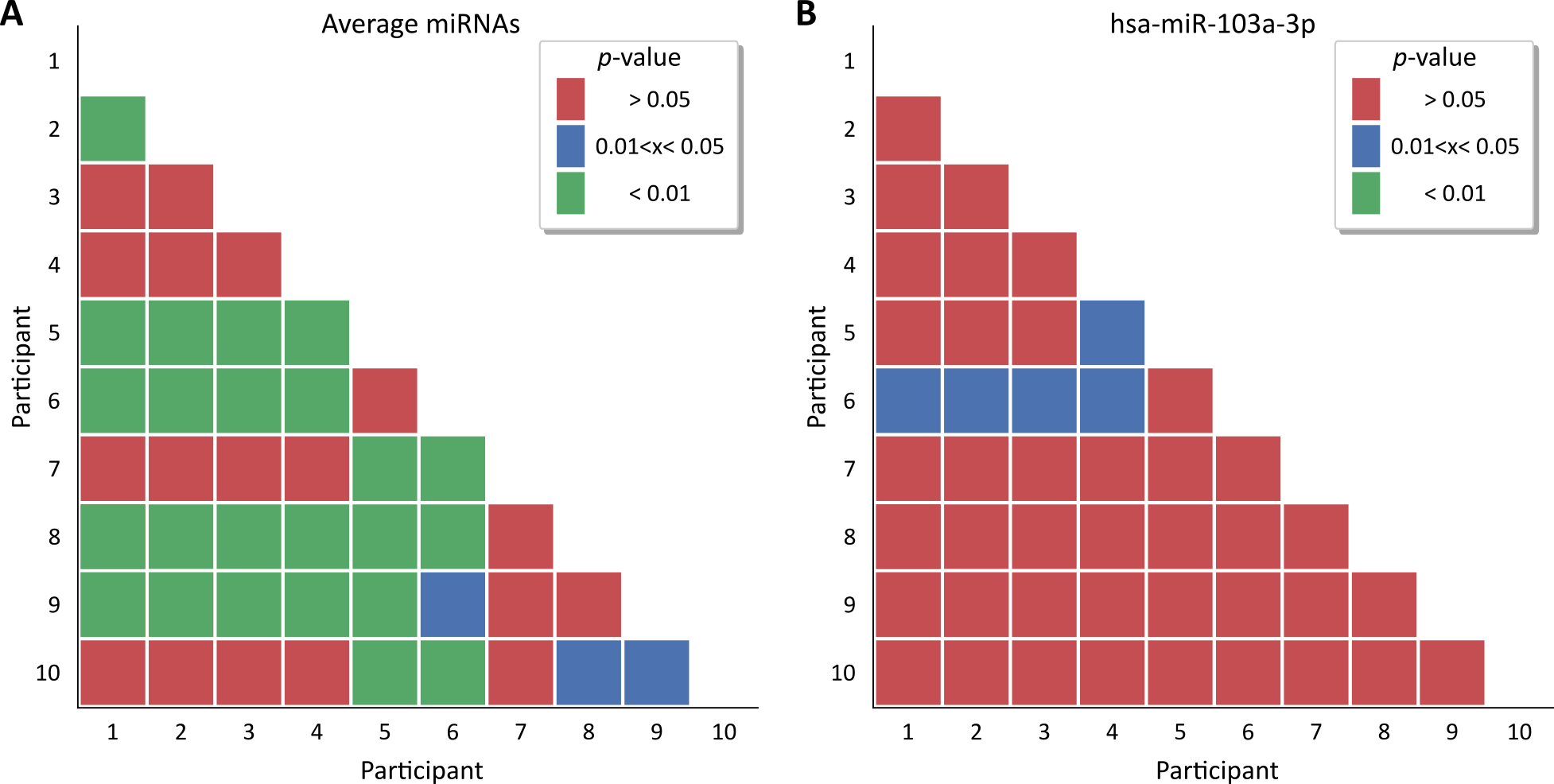

Supplement: S7 Fig — Statistical analysis for data in Fig 2C showing statistical differences within participants (A) when averaging the six miRNAs and (B) within hsa-miR-103a-3p. We note that in panel A, participant P7 and participant P10 do not clearly belong to a group but rather are adjacent to a group, sharing partial significance. (TIF) [file pone.0314733.s007.tif]

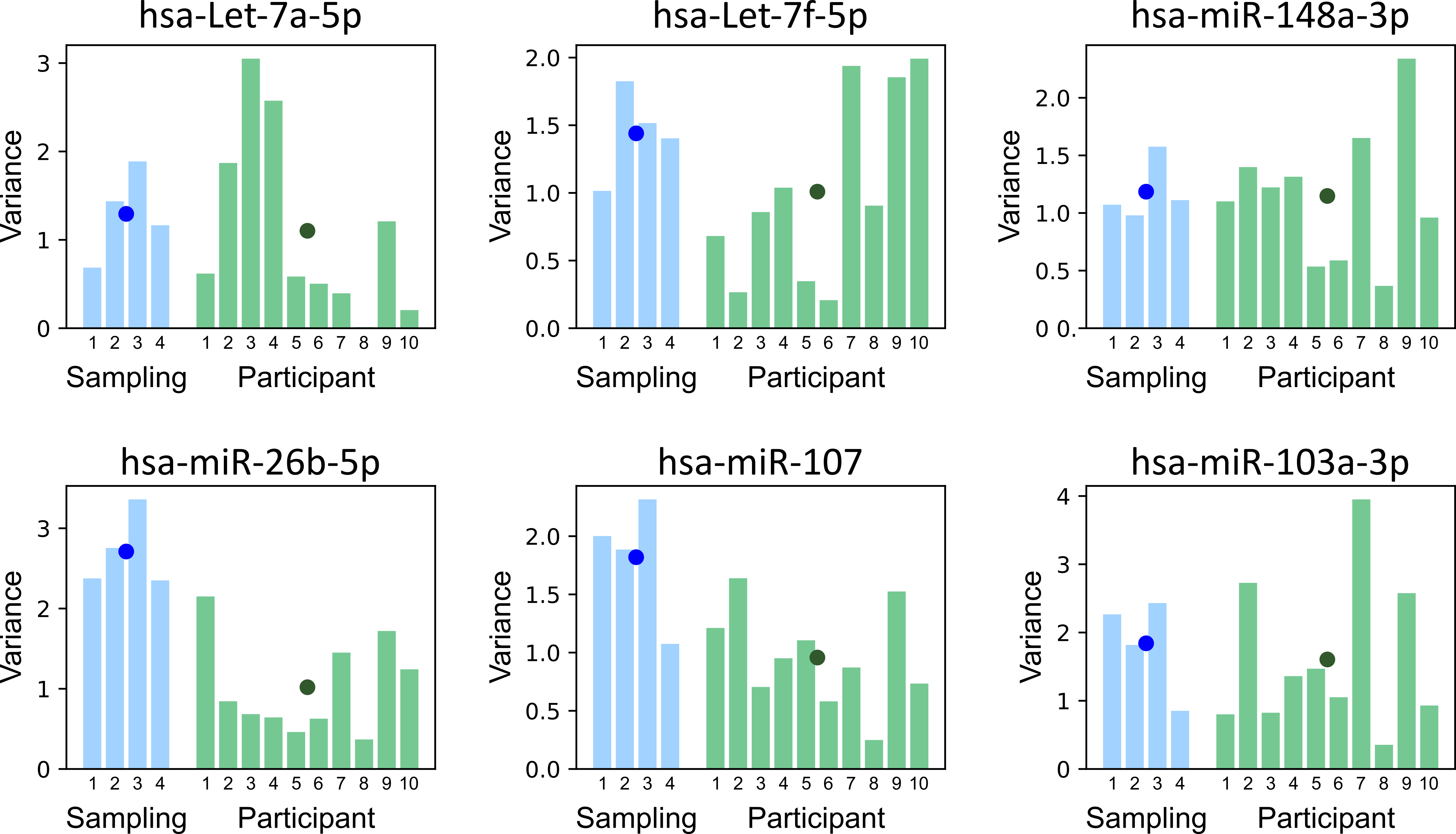

Supplement: S8 Fig — (TIF) [file pone.0314733.s008.tif]

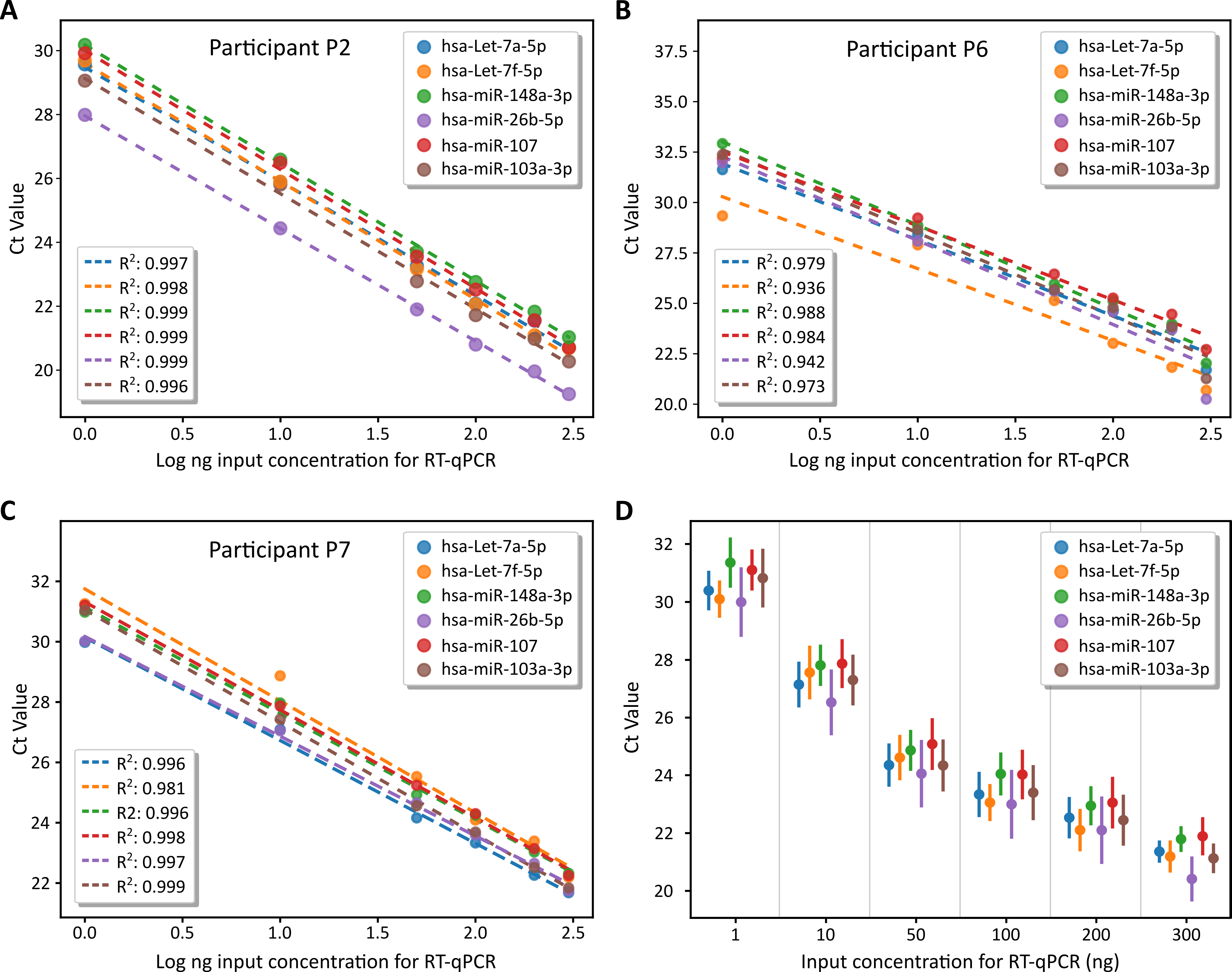

Supplement: S9 Fig — Ct values for participant P2 (A), P6 (B) and P7 (C). (D) Average Ct values of the 3 participants with respect to the miRNA assay at different concentrations of total extracted small RNA. Error bars show the standard error of the mean. (TIF) [file pone.0314733.s009.tif]

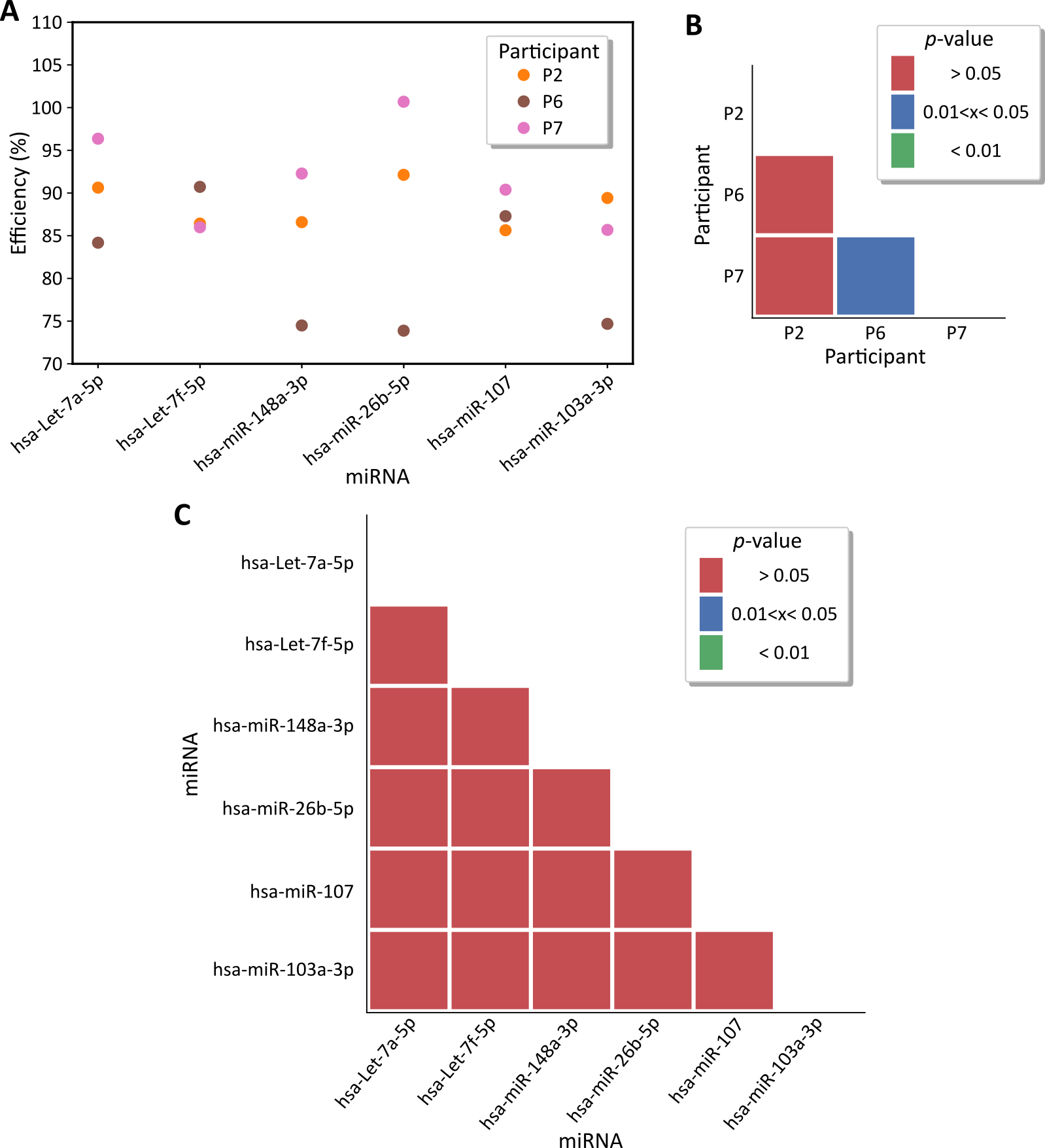

Supplement: S10 Fig — (A) RT-qPCR efficiencies calculated from the dose dependent data presented in S5 Fig. Mann-Whitney U results for panel A with respect to participants (B) and miRNA assay (C). (TIF) [file pone.0314733.s010.tif]

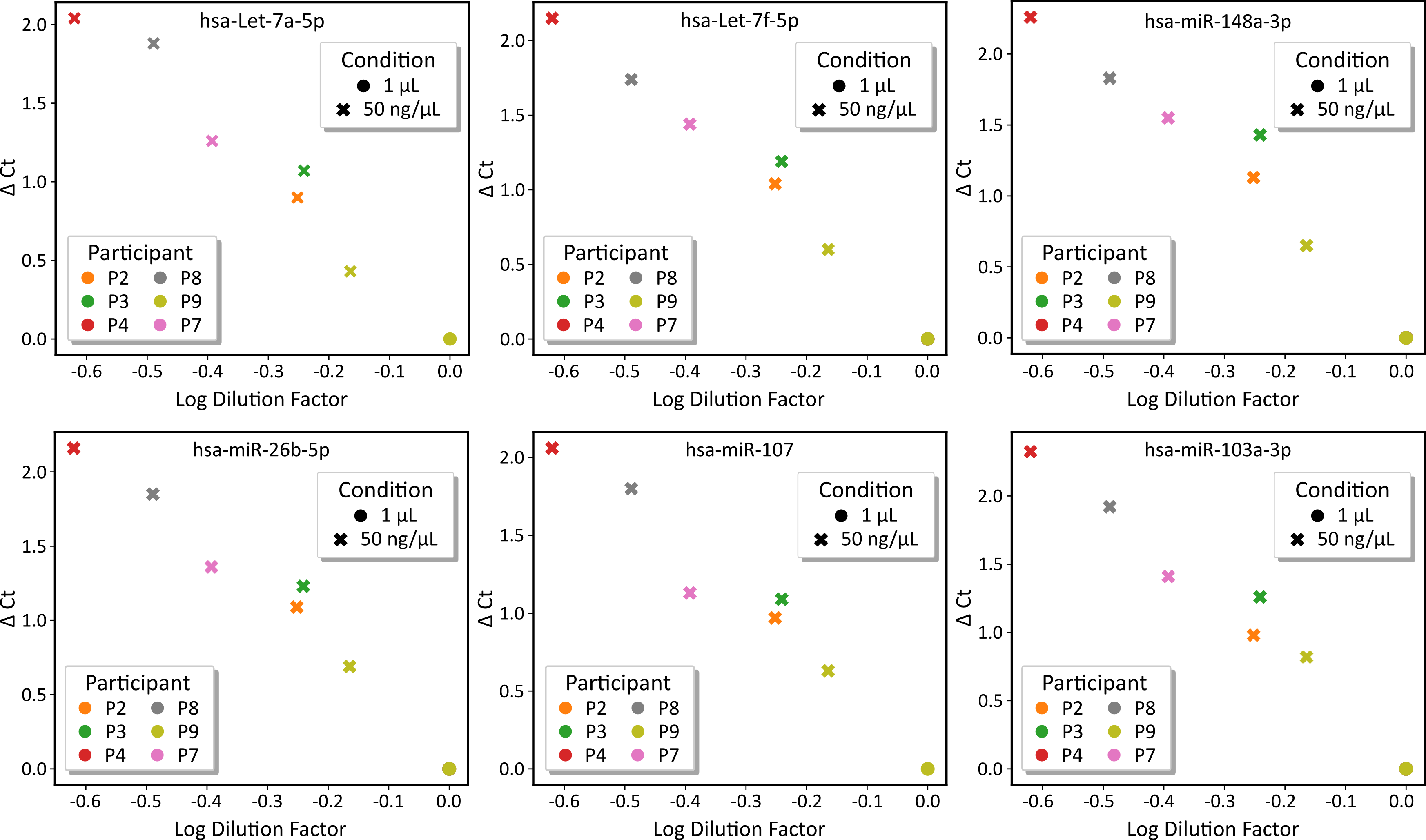

Supplement: S11 Fig — As for S4 Fig, two inputs concentrations where used, 1μL (circle) or 50 ng (cross). Again, all 50 ng values have been normalized with respect to the RNA concentration at 1 μL (dilution factor) and all Ct values have been shifted with respect to the 1 μL value. Although in this case the miRNA concentration may or not be heterogeneous, a linear behaviour is still obtained because we are observing the linear behaviour on the Ct value (ΔCt) due to the dilution effect (log dilution factor). ΔCt values = Ct50ng–Ct1μL. (TIF) [file pone.0314733.s011.tif]

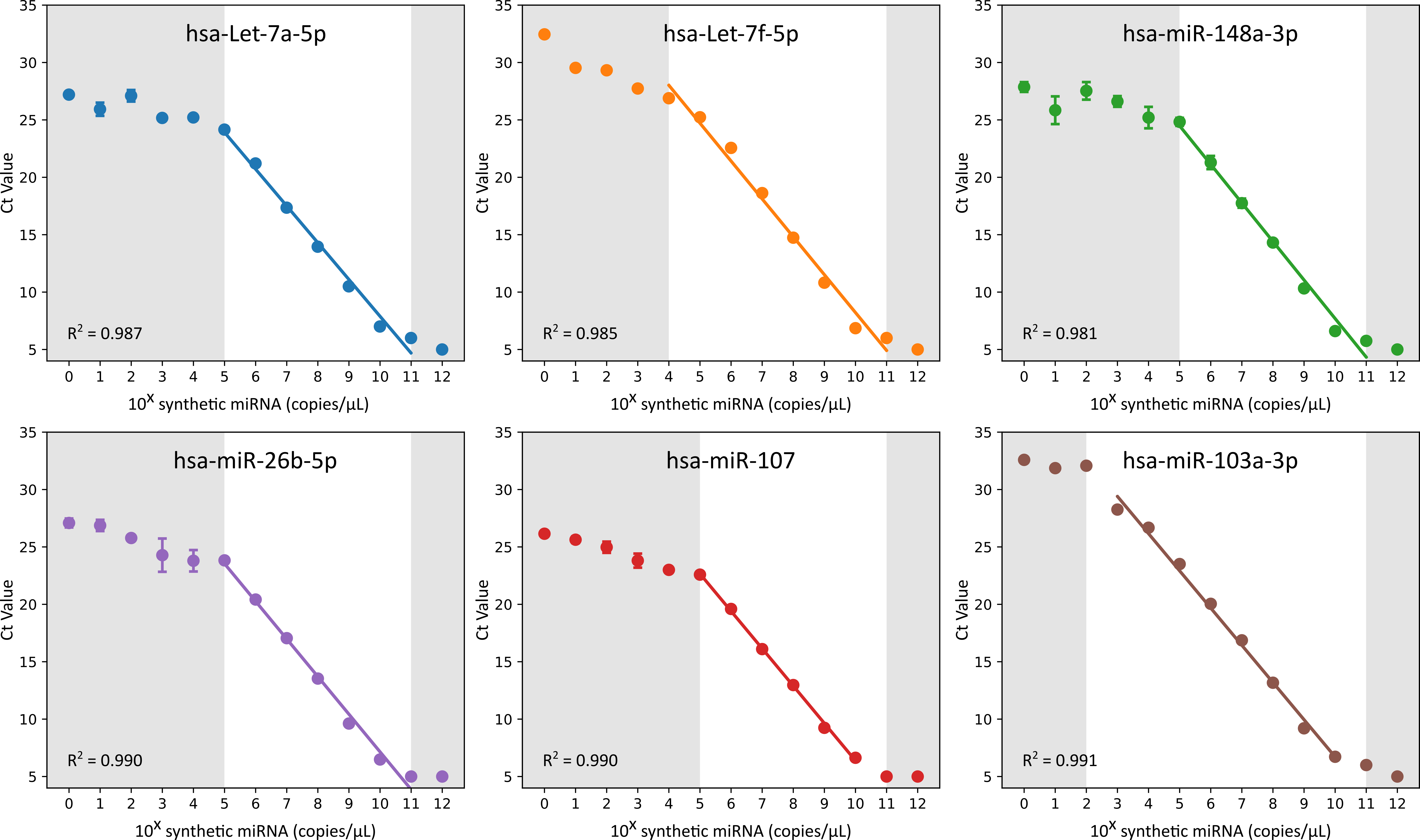

Supplement: S12 Fig — Individual plots for data shown in Fig 3A of the manuscript, demonstrating high sensitivity (LOD of 1 copy/μL) for all six assays. Regression line calculated within LOQ and Ct saturation point (i.e. Ct value = 5). Points represent the mean value and error bars depict the standard error of the mean. The grey region delimits the quantification region. (TIF) [file pone.0314733.s012.tif]

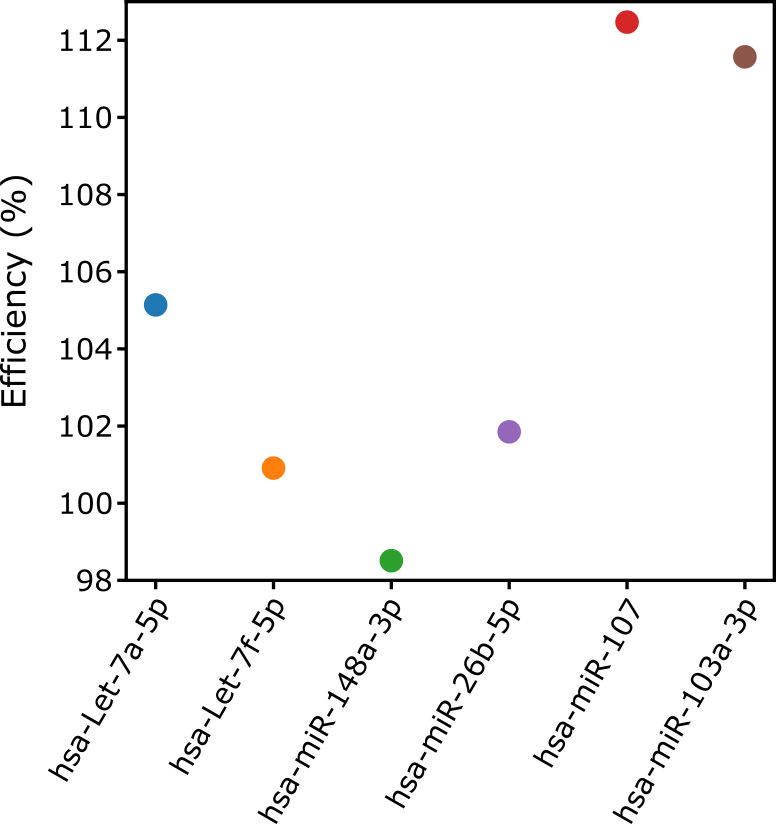

Supplement: S13 Fig — Data from S12 Fig was used to calculate efficiencies. (TIF) [file pone.0314733.s013.tif]

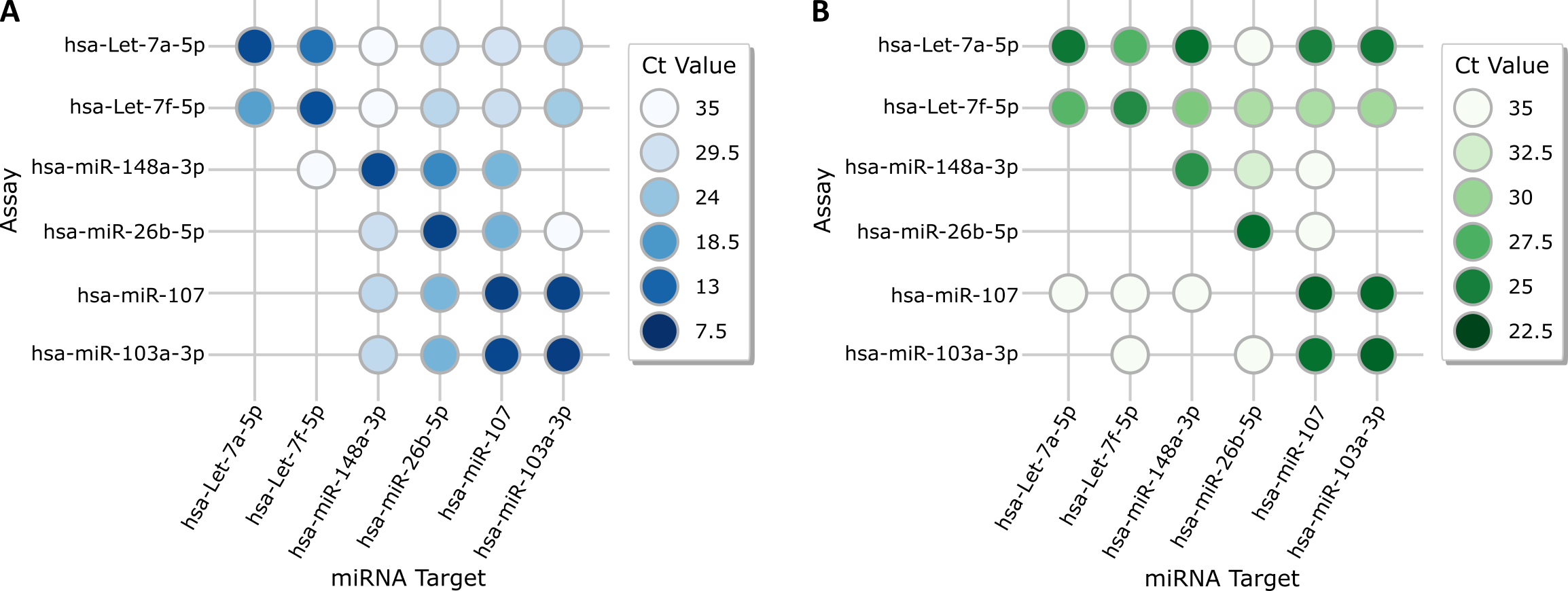

Supplement: S14 Fig — Cross detections among miRNA assays when using (A) 109 copies/μL and (B) 105 copies/μL of synthetic targets. The absence of circles indicate no detection or Ct values = 35. This data was used to calculate the ΔCt values in Fig 4B & 4C of the manuscript. (TIF) [file pone.0314733.s014.tif]

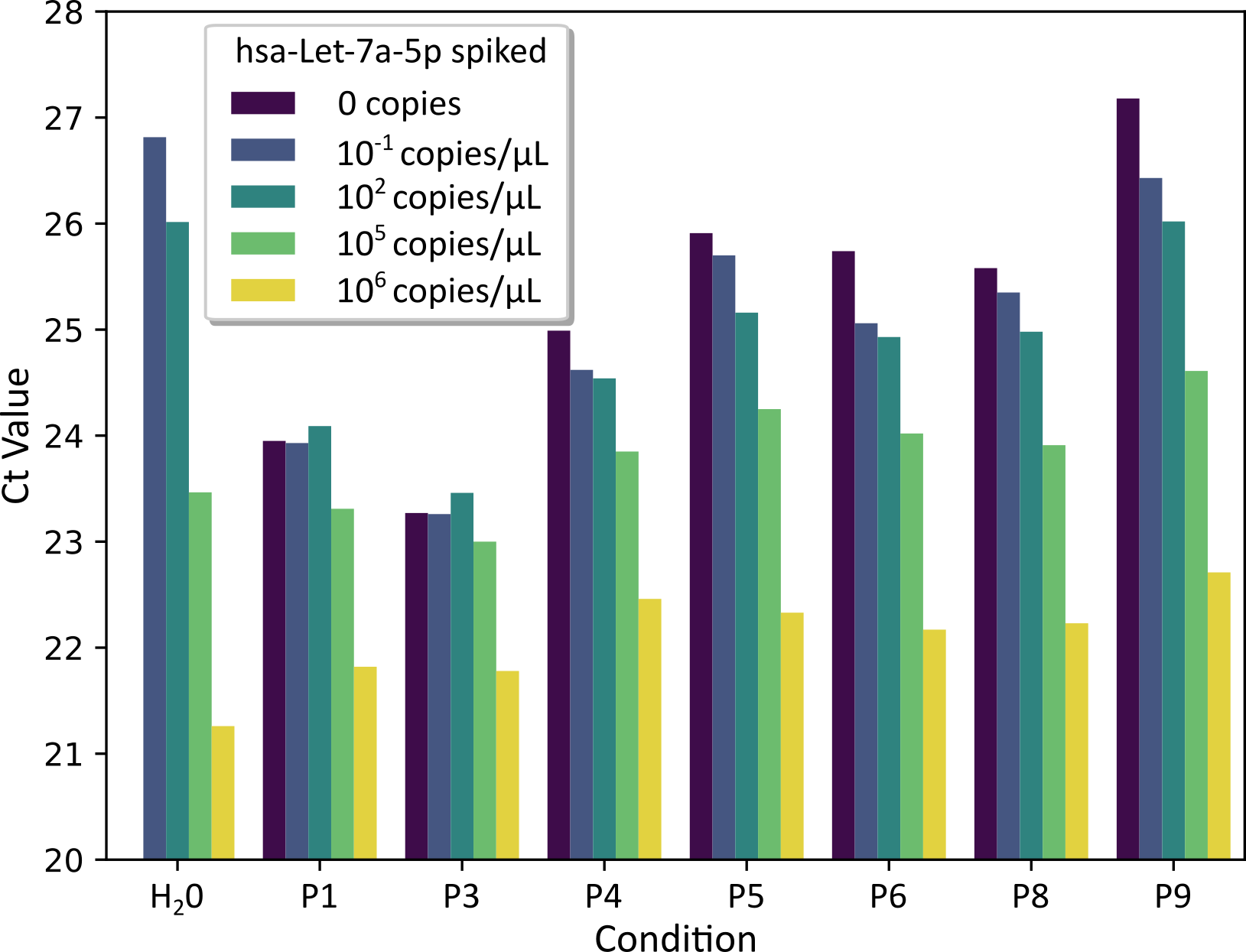

Supplement: S15 Fig — Data used to calculate the ΔCt values in Fig 5 of the manuscript. (TIF) [file pone.0314733.s015.tif]

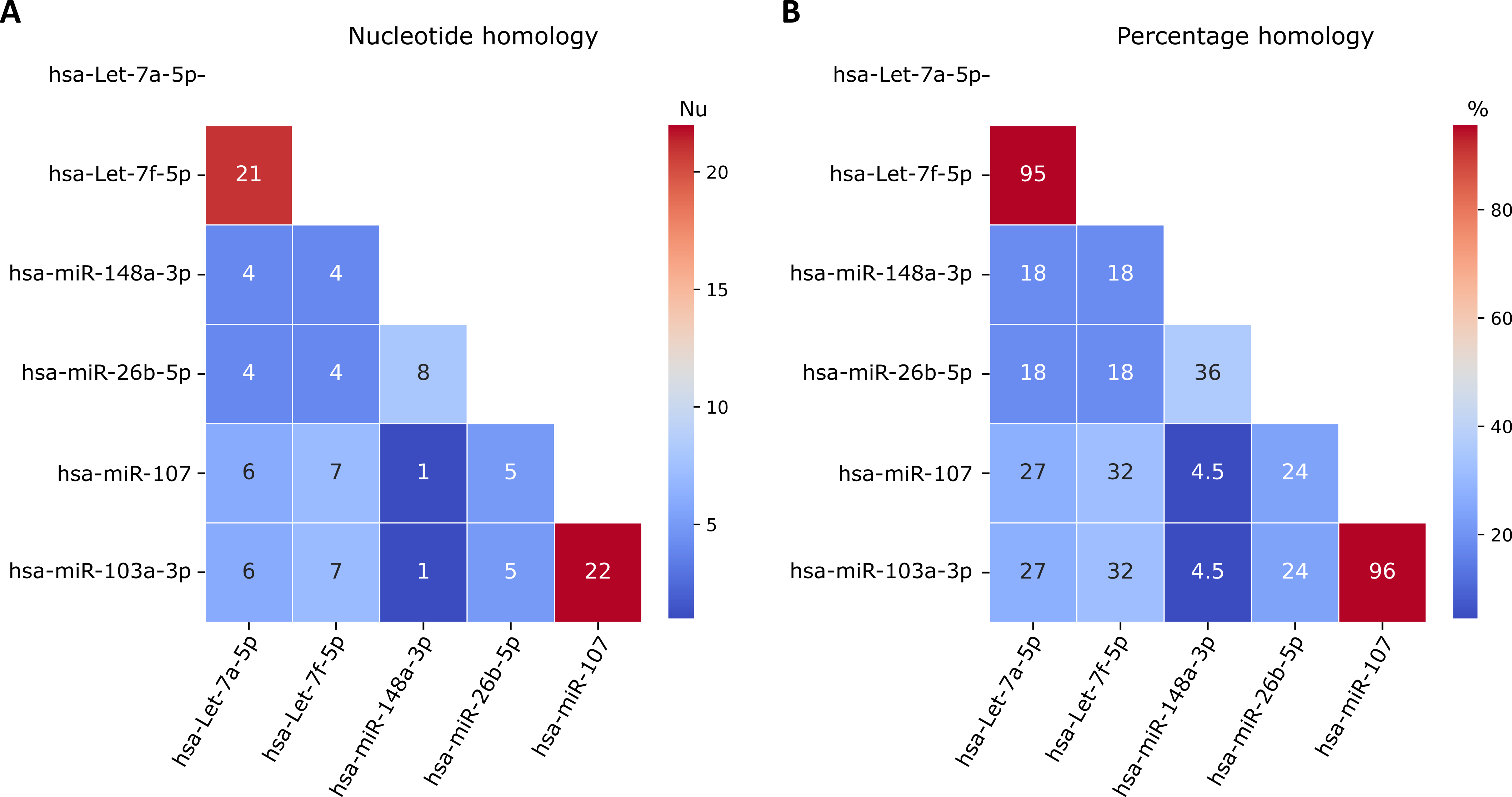

Supplement: S16 Fig — Sequence homology calculated by (A) number of homologous nucleotides and (B) percentage homology. (TIF) [file pone.0314733.s016.tif]
