# Supplementary material for: Assessment of salivary microRNA by RT-qPCR: Facing challenges in data interpretation for clinical diagnosis
Source: PLoS One. 2024 Dec 10;19(12):e0314733. doi: 10.1371/journal.pone.0314733 (PMC11630609; doi:10.1371/journal.pone.0314733)
Supplement: S1 Table — Two similar pairs of miRNAs were included in this study, where they only differentiated by a single nucleotide (red font for the first pair and blue for the second pair). (DOCX) [file pone.0314733.s017.docx]

**Table S1. The miRNAs chosen in this study.** Two similar pairs of miRNAs were included in this study, where they only differentiated by a single nucleotide (red font for the first pair and blue for the second pair).

| No | Accession miRBase | Name of mature miRNA | Sequence |
| --- | --- | --- | --- |
| 1 | MIMAT0000062 | hsa-let-7a-5p | UGAGGUAGUAGGUUGUAUAGUU |
| 2 | MIMAT0000067 | hsa-let-7f-5p | UGAGGUAGUAGAUUGUAUAGUU |
| 3 | MIMAT0000243 | hsa-miR-148a-3p | UCAGUGCACUACAGAACUUUGU |
| 4 | MIMAT0000083 | hsa-miR26b-5p | UUCAAGUAAUUCAGGAUAGGU |
| 5 | MIMAT0000104 | hsa-miR-107 | AGCAGCAUUGUACAGGGCUAUCA |
| 6 | MIMAT0000101 | hsa-miR-103a-3p | AGCAGCAUUGUACAGGGCUAUGA |
