# Supplementary material for: Assessment of salivary microRNA by RT-qPCR: Facing challenges in data interpretation for clinical diagnosis
Source: PLoS One. 2024 Dec 10;19(12):e0314733. doi: 10.1371/journal.pone.0314733 (PMC11630609; doi:10.1371/journal.pone.0314733)
Supplement: S2 Table — (DOCX) [file pone.0314733.s018.docx]

**Table S2. Average RT-qPCR Ct values and the standard error of the mean of the four sampling points for each miRNAs assays assessed on the 10 participants.**

|  | miRNA assay | | | | | |
| --- | --- | --- | --- | --- | --- | --- |
| Participant | hsa-let-7a-5p | hsa-let-7f-5p | hsa-miR-148a-3p | hsa-miR26b-5p | hsa-miR-107 | hsa-miR-103a-3p |
| P1 | 24.79 ± 0.39 | 25.37 ± 0.41 | 25.50 ± 0.52 | 24.10 ± 0.73 | 25.65 ± 0.55 | 25.22 ± 0.45 |
| P2 | 23.62 ± 0.68 | 23.94 ± 0.26 | 24.34 ± 0.59 | 22.16 ± 0.46 | 24.30 ± 1.64 | 23.78 ± 0.83 |
| P3 | 24.10 ± 0.87 | 24.54 ± 0.46 | 25.38 ± 0.55 | 25.07 ± 0.41 | 25.81 ± 0.42 | 25.19 ± 0.45 |
| P4 | 24.37 ± 0.80 | 24.53 ± 0.51 | 25.81 ± 0.57 | 24.38 ± 0.40 | 25.45 ± 0.49 | 24.96 ± 0.57 |
| P5 | 26.32 ± 0.38 | 26.97 ± 0.29 | 27.40 ± 0.37 | 27.46 ± 0.34 | 27.98 ± 0.53 | 27.37 ± 0.61 |
| P6 | 25.55 ± 0.35 | 27.09 ± 0.23 | 27.35 ± 0.38 | 27.37 ± 0.40 | 28.26 ± 0.38 | 27.52 ± 0.51 |
| P7 | 24.38 ± 0.31 | 24.77 ± 0.70 | 26.33 ± 0.64 | 25.51 ± 0.60 | 26.77 ± 0.47 | 25.70 ± 0.99 |
| P8 | 25.69 ± 0.06 | 24.25 ± 0.48 | 26.62 ± 0.30 | 25.95 ± 0.30 | 27.12 ± 0.25 | 26.67 ± 0.30 |
| P9 | 25.88 ± 0.55 | 25.04 ± 0.68 | 27.09 ± 0.76 | 25.67 ± 0.66 | 27.07 ± 0.62 | 26.71 ± 0.80 |
| P10 | 25.06 ± 0.23 | 24.99 ± 0.71 | 25.76 ± 0.49 | 24.61 ± 0.56 | 26.27 ± 0.43 | 25.84 ± 0.48 |
