# Supplementary material for: Assessment of salivary microRNA by RT-qPCR: Facing challenges in data interpretation for clinical diagnosis
Source: PLoS One. 2024 Dec 10;19(12):e0314733. doi: 10.1371/journal.pone.0314733 (PMC11630609; doi:10.1371/journal.pone.0314733)
Supplement: S3 Table — (DOCX) [file pone.0314733.s019.docx]

**Table S3. Dilution factors required to achieve 50 ng for the RT-qPCR reaction for the 10 participants and their 4 sampling points.**

| Participant | 1st Sampling | 2nd Sampling | 3rd Sampling | 4nd Sampling | Average |
| --- | --- | --- | --- | --- | --- |
| P1 | 19.68 | 33.82 | 24.30 | 42.24 | 30.01 |
| P2 | 63.92 | 35.83 | 17.86 | 20.34 | 34.49 |
| P3 | 20.04 | 24.67 | 17.42 | 19.14 | 20.32 |
| P4 | 71.24 | 47.04 | 41.70 | 8.92 | 42.23 |
| P5 | 5.26 | 8.00 | 6.88 | 7.26 | 6.85 |
| P6 | 9.48 | 9.74 | 7.96 | 9.38 | 9.14 |
| P7 | 27.2 | 30.00 | 24.70 | 24.36 | 26.56 |
| P8 | 38.7 | 19.74 | 30.86 | 12.16 | 25.37 |
| P9 | 34.12 | 19.20 | 14.60 | 24.48 | 23.10 |
| P10 | 52.44 | 37.73 | 33.88 | 32.48 | 39.13 |
