# Supplementary material for: Assessment of salivary microRNA by RT-qPCR: Facing challenges in data interpretation for clinical diagnosis
Source: PLoS One. 2024 Dec 10;19(12):e0314733. doi: 10.1371/journal.pone.0314733 (PMC11630609; doi:10.1371/journal.pone.0314733)
Supplement: S4 Table — (DOCX) [file pone.0314733.s020.docx]

**Table S4. Limit of detection (LOD) and limit of quantification (LOQ) for the six miRNA assays used in this study.**

| miRNA | LOD (copies/µL) | LOQ (copies/µL) | Ct at LOQ |
| --- | --- | --- | --- |
| hsa-Let-7a-5p | 1 | 10^5^ | 24.16 |
| hsa-Let-7f-5p | 1 | 10^4^ | 26.89 |
| hsa-miR-148a-3p | 1 | 10^5^ | 24.83 |
| hsa-miR26b-5p | 1 | 10^5^ | 23.83 |
| hsa-miR-107 | 1 | 10^5^ | 22.59 |
| hsa-miR-103a-3p | 1 | 10^2^ | 32.08 |
